# Neurosensory transmission without a synapse: new perspectives on taste signaling

**DOI:** 10.1186/1741-7007-11-42

**Published:** 2013-04-15

**Authors:** Sue C Kinnamon

**Affiliations:** 1 Department of Otolaryngology and Rocky Mountain Taste and Smell Center, University of Colorado School of Medicine, 12700 E 19th Ave, Aurora, Colorado 80045, USA

## Taste cell types and transduction

**Figure 1:**
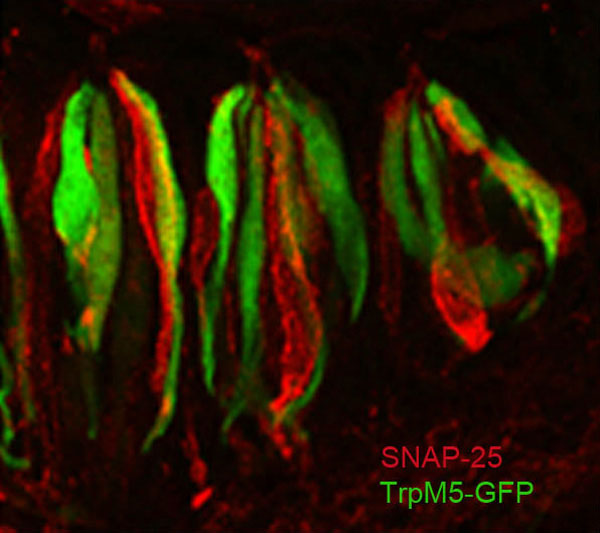
**Circumvallate taste buds contain distinct populations of Type II and Type III taste cells**. Laser scanning confocal micrograph of a longitudinal section through circumvallate taste buds in a transgenic mouse expressing GFP from the TrpM5 promoter, which labels Type II taste cells (green). Type III cells are labeled with an antibody against SNAP-25, a presynaptic protein (red). Taste buds also contain Type I 'glial-like' cells, which are unlabeled and thus not visible. Taste buds consist of 50 to 100 taste cells that are roughly 10 μm across and about 100 μm in height. Taste stimuli contact the apical (top) tips of the cells, while afferent nerve fibers contact the basolateral (lower) portions of the taste cells. Image modified from [2].

Taste buds transduce the chemicals that elicit the sweet, bitter, salty, sour, and umami tastes into membrane depolarization, which triggers release of transmitter to activate gustatory afferent nerve fibers. Each taste bud comprises approximately 50 to 100 taste cells, which can be classified into three distinct cell types based on morphological, molecular and physiological criteria. Type I cells are generally considered to be support cells, similar to glial cells in the nervous system. Their membranes wrap around the other cell types and they express enzymes and transporters for uptake or inactivation of transmitters. Type II cells, also called 'receptor' cells, contain the T1R and T2R families of G protein-coupled taste receptors for bitter, sweet, and umami taste stimuli. Both T1R (for sweet and umami) and T2R (for bitter) receptors activate similar transduction cascades in different subsets of Type II cells. These involve G protein activation of a signaling complex that elicits release of Ca^2+ ^from intracellular stores and subsequent activation of a transduction channel that depolarizes the membrane to cause transmitter release and the activation of gustatory nerve fibers. Type III cells are the sour (acid) transducing cells, and although the transduction mechanisms involved in sour taste have not been clearly elucidated, they likely involve apically located ion channels. These cells are called 'presynaptic' cells, because, in contrast to Type II cells, they exhibit prominent, morphologically identifiable synapses onto afferent nerve fibers and release the neurotransmitters serotonin and GABA in response to sour taste stimuli. Taste cells responsible for salty taste have not been clearly delineated. Figure 1 illustrates how Type II and Type III cells are arranged in taste buds. For review of taste cell types and transduction, see [1].

## Type II taste cells lack voltage-gated Ca^2+ ^channels and the presynaptic protein SNAP-25

In 2006 we reported in *BMC Biology *that Type II cells lack voltage-gated Ca^2+ ^channels and the presynaptic protein SNAP-25-elements normally required for conventional vesicular-mediated synaptic transmission [2]. The 2006 paper followed our previous report showing that although nerve fibers abut Type II cells, these contacts lack specific pre- and post-synaptic specializations that are typical of conventional synapses [3]. Our conclusions, based largely on electron microscopy, immunocytochemistry and electrophysiology of GFP-labeled Type II cells, were confirmed by the molecular studies of another group showing several types of voltage-gated Ca^2+ ^channels in Type III cells, but none in Type II cells [4]. We have extended these initial findings to show that GFP-labeled Type III cells exhibit voltage- and Ca^2+^-dependent increases in capacitance, suggesting vesicular release of transmitter. In contrast, Type II cells do not show capacitance changes typical of vesicular release [5]. If Type II cells lack conventional, vesicular-mediated synaptic transmission, how do they communicate bitter, sweet, and umami taste information to the nervous system? We suggested two possibilities in the 2006 paper: (1) Type II cells communicate with afferent fibers via the agency of Type III cells, possibly via electrical coupling involving gap junctions between Type II and Type III cells; or (2) Type II cells communicate directly with afferent nerve fibers, but via non-vesicular synaptic mechanisms. The first possibility was ruled out by experiments showing that when Type III cells are ablated by selective expression of diphtheria toxin, only sour taste is eliminated - there is no effect on afferent nerve responses to bitter, sweet, or umami stimuli [6]. Hence, Type III cells are not obligatory intermediates between Type II cells and nerve fibers. Thus, Type II cells must signal directly to afferent nerve fibers, likely via a non-vesicular mechanism.

## Taste cells use ATP as a transmitter

A clue to how Type II cells may signal directly to the nervous system came from studies suggesting that ATP is a crucial transmitter for communicating gustatory information to nerve fibers [7]. Evidence for the role of ATP as a primary transmitter in taste buds is based on the findings that mice lacking the purinergic receptors P2X2 and P2X3 (P2X2/3 DKO mice) lack physiological and behavioral responses to most taste stimuli; that taste stimuli evoke release of ATP from taste tissue; and that Type I taste cells contain an ectoATPase for hydrolyzing the released ATP [8]. Since ATP can be released by non-vesicular as well as vesicular mechanisms, particularly in non-neuronal cells, non-vesicular ATP release is a possible mechanism for the release of transmitter from taste cells lacking conventional synaptic machinery. Indeed, several ion channels known to release ATP as a transmitter in other cell types have been identified in taste cells. These include the ion channel pannexin 1, the gap junction hemichannels connexins 43 and 30 [9,10], and a newly identified channel, CALHM1 [11]. Although the exact molecular identity of the ATP release channel is still uncertain, it is clear that ATP is released from taste cells via ATP-permeable channels rather than conventional vesicular-based mechanisms. Evidence for non-vesicular release of ATP from taste cells and the potential role of each identified ATP-release channel is considered below.

## Type II taste cells release ATP by non-vesicular mechanisms

**Figure 2:**
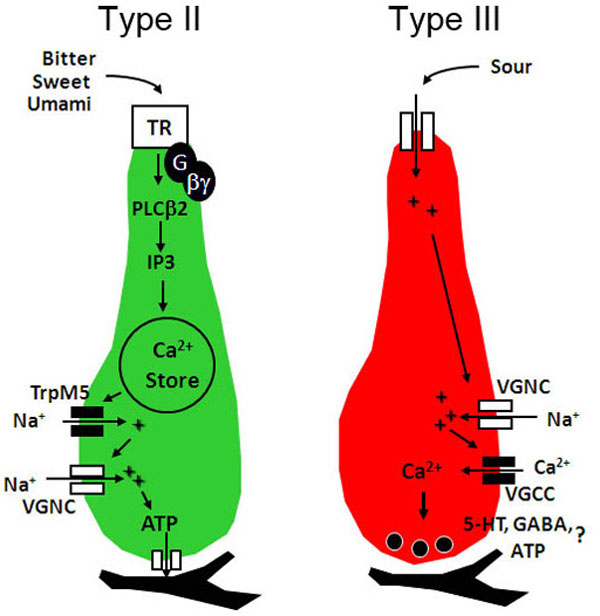
**Type II and Type III taste cells utilize different transduction and signaling mechanisms**. Type II cells (left) contain the G protein-coupled taste receptors (TR) for bitter, sweet, and umami taste stimuli. Although the receptors are expressed in different subsets of Type II cells, they all couple to the same downstream signaling cascade, which includes Gβγ activation of phospholipase C β2 (PLCβ2), causing release of Ca^2+ ^from intracellular stores, Ca^2+^-dependent activation of transient receptor potential cation subfamily M member 5 (TrpM5), membrane depolarization, and release of ATP as a transmitter via an ATP-release channel. Type III cells (right) respond to sour stimuli. While Type II cells signal to afferent fibers by releasing ATP via ATP-permeable channels, Type III cells form conventional synapses and release transmitter via exocytosis. The molecular identity of the ATP release channel and the transmitter composition of the vesicles released by Type III cells at the synapse with the afferent nerve remain important unresolved questions. Other abbreviations: serotonin (5-HT), voltage-gated calcium channel (VGCC), voltage-gated sodium channel (VGNC).

The ion channel pannexin 1 is expressed in all Type II taste cells as well as some Type I and Type III cells, and its expression appears to be restricted to taste buds with little or no expression in surrounding non-gustatory epithelia. Thus, pannexin 1 exhibits the requisite distribution expected for a channel mediating ATP release in taste cells. Physiological evidence for a role of pannexin 1 was suggested by experiments using biosensor cells expressing purinergic P2X receptors. When isolated single Type II cells are exposed to bitter and sweet stimuli, biosensor cells respond with a Ca^2+ ^signal that is inhibited by low concentrations of carbenoxolone, a pharmacological agent reportedly specific for pannexin-based ATP release channels [9]. Further studies have shown that taste-evoked ATP release is dependent on intracellular Ca^2+ ^and the transduction channel TrpM5 [12], and that ATP released from single Type II taste cells (measured by luciferin/luciferase assay) is directly proportional to the number of action potentials evoked by taste stimulation [13]. Collectively, these data suggest a model in which Type II cells are activated by taste stimuli, causing release of Ca^2+ ^from intracellular stores, which activates TrpM5, resulting in depolarization and activation of voltage-gated Na^+ ^channels, which ultimately trigger opening of the voltage- and Ca^2+^-dependent pannexin 1 ATP-release channel (Figure 2).

The role of pannexin 1 as the primary ATP release channel has been seriously challenged by a different group, using a similar biosensor cell assay. They reported that when Type II cells are depolarized by voltage (using voltage-clamp rather than taste stimuli), ATP release is inhibited by Gap26 and octanol, selective inhibitors of connexin-based hemichannels [10]. In those studies, carbenoxolone was without effect, suggesting that pannexin 1 was not involved. Further, using physiological recordings together with biophysical modeling, the authors argue that the kinetics of ATP release from taste cells favors a connexin-based hemichannel over a pannexin-based channel [14]. It is possible that these differences reflect the different modes of stimulation (voltage clamp versus taste), since intracellular Ca^2+ ^is clamped to low levels during voltage-clamp recording and this would favor release via connexin-based hemichannels. However, this same group has recently obtained more compelling evidence, using isolated taste buds from pannexin 1 knockout mice [15]. They showed that both taste stimuli and voltage were able to evoke ATP release from isolated taste buds lacking pannexin 1. At a minimum, these data suggest that pannexin 1 is not required for ATP release, although the knockout mice need to be evaluated at a systems level to determine if the knockout affects taste physiology and behavior.

Evidence for a role of CALHM1 in taste-evoked ATP release comes from a recent study showing that CAHLM1 is exclusively expressed in Type II taste cells; that CALHM1 knockout mice have severely diminished responses to bitter, sweet, and umami taste stimuli; and that CALHM1, when expressed alone in heterologous cells, can mediate ATP release [11]. The requirement of CALHM1 in both ATP release and in signaling to afferent fibers strongly suggests that CALHM1 plays a crucial role in ATP signaling in taste cells, but whether CALHM1 is the ATP release channel or part of a heteromeric channel complex with other ATP release channels is not known. CALHM1 has a unique pharmacological profile, being sensitive to the ion channel inhibitor gadolinium, but insensitive to both connexin and pannexin channel inhibitors. Thus, the pharmacological profile of CALHM1 is not compatible with the previous pharmacological studies of ATP release in isolated taste buds [9,10]. A thorough study of the pharmacology of ATP release in taste cells utilizing inhibitors of CALHM1 may shed light on the precise role of CALHM1 in taste-evoked ATP release. Clearly the molecular identity of the ATP release channel is one of the more compelling questions in the field.

Why should taste cells utilize a non-vesicular mechanism for activation of sensory afferents? ATP signaling is widespread in neurosensory perception, in some cases involving non-vesicular release, but in all cases except taste ATP serves as a modulator, rather than a direct activator of afferent signaling [16]. In those systems, ATP is either a co-transmitter or serves to modulate the effectiveness of the primary transmitter, which is usually glutamate. A possible explanation resides in the fact that taste, unlike other sensory modalities, does not require precise timing for quality discrimination. Further, taste cells have a different embryological origin than other sensory cells, being derived from local epithelium rather than neural crest and sensory placodes [17]. Non-vesicular release of ATP is common in epithelial tissues, but less common in neural tissues.

## Remaining questions

In addition to the molecular identity of the ATP release channel in Type II cells, questions also remain about synaptic transmission in Type III cells. The P2X2/3 DKO mice deficient in purinergic receptors lack physiological responses to all taste stimuli, not just bitter, sweet and umami [7]. Further, we have recently confirmed the requirement of ATP by pharmacological inhibition of P2X receptors *in vivo *(Vandenbeuch and Kinnamon, unpublished work). Thus, purinergic transmission is necessary for responses to sour and salty stimuli, as well as the modalities mediated by Type II cells. But what is the source of the ATP for sour and salty stimuli? ATP release has not been detected from Type III cells with either biosensors [9] or luciferin/luciferase assay [13], although if release from these cells is mediated via vesicles rather than channels, it might not be at high enough levels to be detectable using these assays. Another big question is what might be the role of serotonin and GABA in synaptic transmission? Both transmitters are believed to be involved in modulating ATP release from Type II cells [1], but could they also be involved, along with ATP, in activating gustatory afferent nerve fibers? Clearly the next decade will likely bring answers to these compelling puzzles in taste physiology.

## Note

This article is part of the BMC Biology tenth anniversary series. Other articles in this series can be found at http://www.biomedcentral.com/bmcbiol/series/tenthanniversary.
